# Black Women in Medical Education Publishing: Bibliometric and Testimonio Accounts Using Intersectionality Methodology

**DOI:** 10.1007/s11606-024-09117-7

**Published:** 2024-10-23

**Authors:** Witzard Seide, Lauren A. Maggio, Anthony R. Artino, Todd Leroux, Abigail Konopasky

**Affiliations:** 1 Uniformed Services University of the Health Sciences, Bethesda, USA; 2 University of Illinois College of Medicine at Chicago, Chicago, IL USA; 3 George Washington University School of Medicine and Health Sciences, Washington, DC USA; 4 Stonewall Analytics, Eagle, ID USA; 5 Geisel School of Medicine at Dartmouth, Hanover, NH USA

**Keywords:** medical education, Black women, scholarly communication, intersectional/ity

## Abstract

**Background:**

Black women in academic medicine experience racial and gender discrimination, all while being tasked with improving a flawed system. Representation of Black women in medicine remains low, yet they bear the burden of fostering diversity and mentoring trainees, exacerbating their minority tax and emotional labor, and negatively impacting career progression.

**Objective:**

To complement qualitative accounts of Black women authors in the medical education literature with a quantitative account of their representation. We used statistical modeling to estimate the representation of Black women authors in medical education publishing as compared to other groups.

**Design:**

An intersectional methodology employing bibliometric analysis and testimonio reflection.

**Subjects:**

US-based authors of journal articles published in medical education journals between 2000 and 2020.

**Main Measures:**

Author race was determined using a probability-based algorithm incorporating US Census data, and author gender was ascribed using Social Security Administration records. We conducted two negative binomial generalized linear models by first and last author publications. Metadata for each article was retrieved from Web of Science and PubMed to include author names, country of institutional affiliation, and Medical Subject Headings (MeSH). Results were contextualized via the “testimonio” account of a Black woman author.

**Key Results:**

Of 21,945 unique authors, Black women (and other racially minoritized groups) published far fewer first and last author papers than white women and men. In addition, major MeSH terms used by Black women authors reveal little overlap with highly ranked medical education topics. The testimonio further narrated struggles with belonging and racial identity.

**Conclusion:**

This study revealed that Black women are underrepresented in medical education publishing. We believe that dismantling oppressive structures in the publishing ecosystem and the field is imperative for achieving equity. Additionally, further experiential accounts are needed to contextualize this quantitative account and understand underrepresentation in medical education publishing.

## INTRODUCTION

To be a Black woman in academic medicine is to experience discrimination and otherness at the intersection of race and gender,^1^ “drowning in the same system we are burdened to improve.”^2 p^^.1^ This unique location of being Black and a woman is not merely additive (Black + woman), but multiplicative (Black × woman), making it difficult to disentangle their experiences of racism. Yet, Black women are called upon to mend this broken system, by recruiting and mentoring trainees of color and leading diversity efforts; this minority tax burden,^2^ in addition to requiring that Black women perpetually navigate the emotional labor to “anticipate and deflect harm,”^3^^(p329)^ is heavy, since Black women comprise only 1.5% of medical school faculty.^4^

Some Black women scholars are starting to speak out, specifically about their experiences of discrimination in academic publishing, a critical component of academic career progression.^5–7^ For instance, comments from reviewers may express bias when authors reveal their identity,^6^ and this can lead Black women authors to feel like their story is “not good enough.”^5 p^^.145^ Statements from editorial leaders in medical education support these accounts, noting that bias is baked into the publishing process.^8,9^ This evidence argues for the experiential significance of publishing discrimination, but as a field steeped in quantitative methodologies and a postpositivist desire for generalizability, statistical significance is often considered an important aspect of arguing for change.^10^ Regarding gender, quantitative methods have been applied in publishing writ large^11−14^ and medical education specifically,^15,16^ demonstrating unequal patterns for women and men authors (e.g., women tend to write about people; men about power and politics).^11–14^ Meanwhile, regarding race, quantitative methods have found disparities between Black and white authors (e.g., the former lag behind on citations) in scientific publishing,^17–19^ but that work has not yet been done in medical education to our knowledge.

The purpose of this study is to address the gap in intersectional research in medical education publishing. Drawing on the methods of an innovative quantitative study of intersecting inequalities in scientific publishing,^17^ we offer a statistical account of Black women in medical education publishing to complement existing experiential accounts, contextualizing this account through the experiences of a Black woman author in medical education.

### THEORETICAL FRAMEWORK/APPROACH

**Figure 1:**
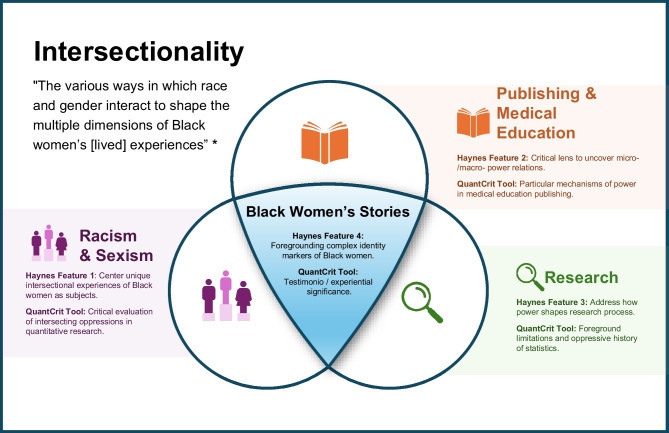
The complexity of intersectionality for Black women in medical education publishing. Note: This figure shows the complexity of intersectionality for Black women in medical education publishing and Haynes’ intersectional methodology (IM) and tools from QuantCrit work as our approach to understanding this complexity. At the core of this approach are Black women’s stories, hence our foregrounding of Black women’s complexity through our authorial testimonio. Then, there are three intersecting sets of investigative tools that we use: first, we focus on the intersectionality of racism and sexism as it applies to Black women, making them the core of our analysis; second, we attend particularly to the mechanisms of power in publishing and medical education, providing context for these mechanisms in our introduction and conclusion; third, we also surface research and its history of oppressive practices as another aspect of our context, foreground past oppressions so that readers interpret the current study’s findings with that history in mind.

Most accounts of publication inequities examine racism and sexism separately. These accounts miss the complex “intersectionality” first noted by Kimberlé Crenshaw, who pointed out the reciprocal and multiplicative functioning of racism for Black women, “shaping structural, political, and representational aspects of violence.”^20^^(p1244)^ (See Fig. 1 for a definition of intersectionality.) In quantitative accounts, narrating this complexity is difficult for several reasons. First, statistical methods rely on separate variables and robust numbers and, as noted above, there are few Black women in academic medicine^4^ and, thus, a small pool of potential medical education authors to form robust numbers. Second, assumptions of objectivity and generalizability work against explaining the contextualized and lived experiences of Black women.^21,22^

#### Haynes’ Intersectionality Methodology

Nonetheless, quantitative approaches can be used in critical ways,^23^ offering a different perspective that can complement, rather than supplant, experiential accounts. Moreover, publications are central sources of advancement and prestige.^5–7^ Therefore, understanding their (in)equitable distribution is critical to making change. To do this, we take Haynes’ intersectionality methodology (IM), developed by Black women scholars through analysis of intersectional studies of Black women, as our theoretical framework (the first use of this approach that we know of in medicine; see Fig. 1).^24^ IM emphasizes four features: (1) centering the unique intersectional experiences of Black women as the subject; (2) use of a critical lens to uncover micro- and macro-power relations (which, crucially, involves recognizing and naming the individual and systemic inequities, something we do throughout the paper); (3) addressing how power shapes the research process, with an emphasis on reflexivity (reflecting on the researcher’s role in the research); and (4) foregrounding complex identity markers of Black women, “presenting Black women in the fullness of their humanity.”.^24^^(p778)^

We address features 1 and 4 below, but we address the others here. IM asks researchers to critically attend to power relations, addressing how they are at work in the research process (IM features 2 and 3). Medical education is dominated by whiteness, which acts as a gatekeeper to keep Black women and others out.^25^ This is evidenced by Black women’s accounts of oppression and exclusion in academic medicine (within which most medical education occurs)^1,2,26,27^ and publishing.^5,6,28^ This power is also evident in the field’s reliance on biomedical inquiry, with its sole focus on the body to the exclusion of political and social structures.^29^ For instance, the term “underrepresented in medicine” both denotes racially minoritized physicians and trainees like Black women as “other” and homogenizes their experiences.^30^ The statistical account detailed below is inextricably tied to this context of power. Moreover, quantitative methodology itself was born out of a racial power imbalance: two of its founders, Galton and Pearson, developed statistical methods largely for eugenics research.^31^ Bonilla-Silva and Zuberi argue that the subsequent history of statistics—up to and including our practice today—has continued to talk causally about the “effect of race,” focusing on the individual rather than the social structure and leading to justifications for racial inequality.^32^

As part of this wrestling with power, we further note that our research team identities reflect power inequities. Indeed, the first author, a researcher with an MD who is a uniformed services officer, is the only Black woman (or Black) member of our team and, as such, the only one with intersectional experiences. The four other authors include two white women and two white men, all PhD-level researchers who have benefited from racism. This imbalance reflects the structural inequities of the medical education research field in which we met, but also reflects how the white academics on the team have not necessarily seen or valued their Black women colleagues appropriately. While we used IM and reflexivity to continually recenter the first author, the first author herself intentionally ensures she is empowered and that her voice is heard throughout this manuscript, we know that the results below are limited by the racial lenses of the four white team members.

#### QuantCrit

We also draw tools from the QuantCrit movement: an application of critical race theory specifically to quantitative analysis.^21^ This movement holds that if researchers use quantitative methods to understand racism, they must use those numbers to work for social justice and must acknowledge the centrality of racism, the subjectivity inherent in numbers (just as much as words), and the fact that neither categories (e.g., Black, woman) nor data (e.g., numbers, *p*-values) are ever “neutral”.^21^

In keeping with QuantCrit approaches, we present some of our limitations here rather than at the end of the paper, highlighting the inequity of our data sources (discussed in more detail below). First, bibliometric tools like the Web of Science (WoS) are created predominantly by male peer reviewers^33^ and white editorial boards.^34^ Second, the US Census was created in part to protect white individuals^35^ and still retains limitations of that history. Finally, the Social Security Administration (SSA) has a racist history, with arguments that it disproportionately benefits whites.^36^

## METHODS

This study took an IM approach, drawing as well on strategies from QuantCrit.

### Centering Black Women in a Quantitative Bibliometric Analysis Drawing from QuantCrit

We conducted a bibliometric analysis^37^ of US-based journal article authors focused on their predicted gender and race/racism, centering Black women (IM feature 1).^24^ This work is a sub-analysis of a larger project that broadly investigated author characteristics around the globe; that prior work did not attempt to identify race/racism.^16^ In the current study, we utilized a subset of the data specific to US-based authors. The data set drew from the Medical Education Journal List (MEJ-24), described as a “seed set of journals” representing the field of medical education.^38^ The MEJ-24 was derived using co-citation, an evidence-based, bibliometric approach for field delineation (while also created largely by those in positions of publishing power).^39–41^ All data assembly, cleaning, and statistical modeling were performed using R version 4.2.1.

While we seek to support experiential accounts of Black women with a statistical account, we recognize the bibliometric and statistical methods we use are themselves oppressive, steeped in the white logic of the academy.^21,42^ We draw four tools from QuantCrit^21^ to mitigate this: (a) whenever possible (i.e., when it will not be misrepresentative), we foreground intersectional identities;^10^ (b) we always refer to racism alongside race; (c) we contextualize our work around power and reflexivity (see sections above);^10,23^ and (d) our first author offers a *testimonio*—“purposeful storytelling grounded in praxis utilized to expose and disrupt histories that are otherwise subsumed”^10^^(p255)^—to center lived experience (IM feature 4). This testimonio is not intended to be “generalizable,” but instead, “rooted in oral history and human rights struggles, testimonio is purposeful storytelling grounded in praxis utilized to expose and disrupt histories that are otherwise subsumed.”^10^^(p255–256)^

#### Data

On August 27, 2021, we used WoS to retrieve metadata for articles published in 22 of the MEJ-24 journals between 2000 and 2020 (as the *Journal of Graduate Medical Education* and the *Canadian Medical Education Journal* are not indexed in WoS). On the same day, we downloaded metadata for all MEJ-24 journals using the Crossref REST API. Metadata included author first and last names, journal, publication year, title, and abstract. For all journals, we downloaded the associated Medical Subject Headings (MeSH), which we use as a proxy for article topic (recognizing the limitations from its origination in the oppressive structure of publishing). From this data set, we identified and extracted all authors affiliated with institutions in the United States (US). We necessarily focused on US-based authors because our approach utilizes US Census data to provide the basis for making estimations on author race/racism. Two separate algorithms were utilized to provide estimates on race/racism and gender for the authors captured in these journals. In keeping with IM, we present and interpret our findings as an *intersection* of these algorithms.

#### Race/Racism and Ethnicity Algorithm

We adapted a race/racism algorithm originally developed by Kozlowski et al.,^17^ which utilized family names (surnames) and given names, with data on frequency counts by the US Census Bureau, to assign probabilities of race/racism based upon these names. The algorithm collapsed categories of Asian*, Black*, Hispanic* (an ethnicity but we used it alongside racial categories due to how it is used in the Census and the effect ethnicity also has on discrimination and oppression), white, and other* (these categories are not necessarily reflective of the lived identity of those to whom they refer, but we use the Census terms in order not to misrepresent the data set; we henceforth mark them with an asterisk to note their potential to oppress). We collapsed some race/racism groupings from the 2010 Census to increase power for estimating effect sizes. Of note, two categories were pooled (“Other*”) when performing summary statistics and statistical models due to low sample sizes (Non-Hispanic American Indian* and Alaska Native Alone* and Non-Hispanic Two or More Races*).

A key element of this algorithm is that, rather than utilizing a race/racism assignment of authors to the most prominent racial group, each author was given an associated *probability* to each race/racism classification (i.e., the sum of all race/racism categories, by author, sums to 100%). Previous research demonstrated^17^ that assigning race/racism based upon the most prominent racial group (or an overall attribution) often underestimates Black* authors and overestimates white authors. We used this algorithm with assigned probabilities across all racial categories to examine intersectionality at the aggregate level, not the individual author level. In cases of missing data, if the Census data did not capture the family and given name, mean imputation was used for the author’s racial probability based upon the distribution for the entire analytic sample.

#### Gender Algorithm

While gender is neither fixed nor binary, it is a powerful social construct, so we used the categories “women” and “men” here to explore sexism. To classify authors by gender, given name data covering years 1880–2021 from the SSA^43^ were used to assign gender categories to authors based upon the frequency of the name from birth records. These data captured the top 1000 names for each year, and each name is associated with a binary gender (women or men). Unlike the above racial attribution methodology, here, authors were classified as women or men according to which name in the SSA data had the greatest frequency attributed to the specific gender rather than assigned a probability.

#### Statistical Analysis

Two negative binomial generalized linear models were used to estimate the representation of Black* women in medical education publishing as compared to other groups, from 2000 to 2020, by both first author publications and last author publications. For both negative binomial models, the outcome variable was the sum of manuscripts published by each race/racism and gender category by publication year. Indicator variables were then constructed for time and to identify Black* authors and women authors. More specifically, the reference categories were men and all other races, centering Black women. When examining the statistical model results, the “Black*” and “women” labels correspond to the average difference as compared to the reference groups.

#### MeSH Analysis

To evaluate first authors’ article topics, we paired major MeSH with the first author publications. Percentile rankings for each racial category and associated “feminization” (i.e., higher proportion of women) were calculated using the joint probability for race/racism and the proportion of women authors for individual terms, respectively.

## RESULTS

The sample consisted of 21,945 unique authors. Of these authors, 43% (*n* = 9553) were represented as first authors and 37% (*n* = 8280) as last. For first authors, 5.6% (*n* = 541) had missing race/racism values imputed. For last authors, 6.6% (*n* = 550) had missing race/racism values imputed. Just under 5% (*n* = 1090) of authors could not be assigned a gender as their given name was absent from the SSA data.

The first step toward our intersectional analysis was examining the descriptives for race/racism and gender separately from papers published between 2000 and 2020 (see Table 1). Black individuals made up only 9% of the sample and women represented 49.9% (excluding non-attributable sources).

**Table 1:** Summary Statistics on Unique Authors (*n* = 21,945)

Category	Statistic
Black*, mean (SD)	0.090 (0.126)
Asian*, mean (SD)	0.132 (0.276)
Hispanic*, mean (SD)	0.061 (0.156)
Other*, mean (SD)	0.024 (0.021)
White, mean (SD)	0.693 (0.299)
Women, % (*n*)^*^	44.9% (9372)

^*^Gender could not be assigned to 1090 authors, so the total sample for this category is 20,855

**Figure 2:**
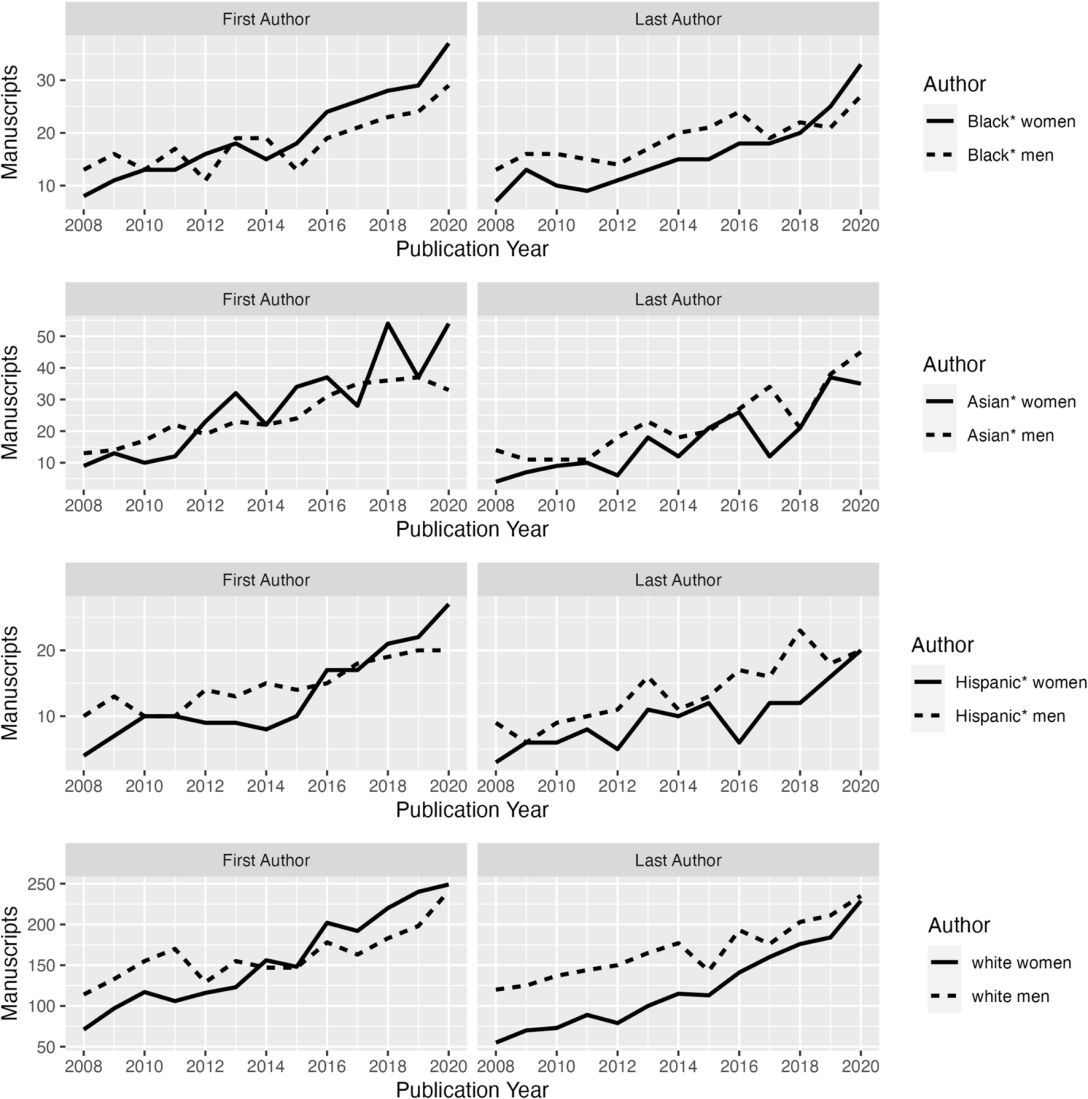
Extrapolated manuscripts by author type, gender, and race/racism. Note: The *y*-axes in the faceted plots have free-floating scales.

Moving to an intersectional lens, Fig. 2 presents the extrapolated manuscript counts by author type. Of note, the *y*-axis has a “floating” (not fixed) scale, which could mislead the reader into thinking that the magnitude of publications across races/racisms is similar. It is not white women and men have far more publications, followed by Asian* women and men, then Black* women and Black* men, and then Hispanic* women and men. We used this floating scale to highlight the trends for Black* women compared to others and to avoid “drowning out” by white authors. As Fig. 2 shows, Black* women have far fewer first and last author publications than white women and men. Looking within races, regarding first authorship, Black* women have had higher counts than Black* men since 2015. Similarly, Hispanic* women and Asian* women first authors have larger counts than Hispanic* men and Asian* men respectively. Regarding last authorship, Black* women moved ahead of Black* men in 2018 and Hispanic* women, Asian* women, and white women last authors remain behind Hispanic* men, Asian* men, and white men.

The negative binomial generalized linear model in Table 2, however, shows that the difference between Black* women and Black* men is not statistically significant in either the first or last author model. When examining Black* women and men together, however, there is a significant and large difference: the average incident rate ratio (IRR) among first and last Black* women and men authors, as compared to all other authors, is 66% lower and 64% lower, respectively. In other words, the average publication rate across time decreased by approximately 65% for Black* women and men authors compared to white authors. In both models, the function of time was statistically significant, indicating that the average rate of published manuscripts increases over time.

**Table 2:** Negative Binomial Author Model Results

Coefficient	First author model			Last author model		
	IRR	SE	*z* value	IRR	SE	*z* value
(Intercept)	29.90***	0.22	14.80	28.30***	0.23	14.2
Black* × woman	1.11	0.47	0.21	1.16	0.48	0.30
Black*	0.34**	0.33	− 0.31	0.36**	0.34	− 2.93
Woman	0.92	0.20	− 0.37	0.68	0.21	− 1.76
Time	1.08**	0.02	3.08	1.09**	0.02	3.27

^***^p-value < 0.001; ***p-value < 0.01; *p-value < 0.05. *IRR*, incident rate ratio; *SE*, standard error

Regarding topics, the top 10% of major MeSH terms for first authors with greater than 60% feminization and exceeding the 50th percentile ranking in Black* authors (i.e., highly likely Black* women) were career mobility, cultural diversity, cooperative behavior, manikins, writing, fellowships and scholarships, empathy, internet, and job satisfaction. Comparing these to the top major MeSH terms of all authors (Table 3), there appears to be little overlap.

**Table 3:** Top 10 Ranking of Overall Medical Subject Heading (MeSH) Terms in Analytic Sample

Major MeSH term	*n*	Proportion of total MeSH terms
Education, Medical	1800	0.105
Clinical Competence	896	0.052
Curriculum	657	0.038
Students, Medical	632	0.036
Faculty, Medical	335	0.01
Learning	312	0.01
Attitude of Health Personnel	273	0.01
Schools, Medical	227	0.01
Career Choice	217	0.01
Physician–Patient Relations	217	0.01

Proportion values will not sum to 1 due to rounding

### Testimonio

To contextualize the above statistical account, the first author shared her lived experiences through a testimonio.

#### Background: A Little Piece of Haiti

My crowded childhood home in Queens, NY, was a little piece of Haiti. The sounds of Haitian Creole and smells of Haitian food were steeped into my identity as a Black woman. My parents raised us to value education and worked hard to provide for us. They expected us to get straight A’s and take up a “respectable” profession: lawyer, doctor, engineer, or nurse.

Captivated by my parents’ vision of success and work ethic, I pursued becoming a doctor. This far-fetched dream to lead myself out of hardship was supported by my family and community who affectionately called me “doc” from the age of 10. Despite my scholarly accomplishments, school counselors routinely recommended that I adjust my goals to be more “realistic,” but I was determined and went on to vocational high school for nursing, thinking this path would offer financial support alongside exposure to medicine. An unexpected full-ride scholarship to a university was my ticket out of Queens, but I planned to return and practice medicine within the medically underserved community I grew up in. After considering finances alongside my goals, I eventually pursued a military medicine scholarship. After my Army service obligation as a pediatrician, I eventually transitioned to opportunities allowing me to have a greater impact on underserved communities. Even with these accomplishments, I never forgot my Haitian roots and occasionally feel guilty as one of the few to “make it out.”

#### Training and Early Career: Struggling to Belong

The path to medicine was fuzzy to me, particularly without any physician mentors. My limited knowledge of the premedical path presumed bench research was needed, so I struggled through that, eventually turning in my pipette for a stethoscope in medical school.

Residency was challenging, with several experiences that left me questioning if I belonged. But determined to succeed, I tirelessly sought feedback and worked hard on regaining confidence after what felt like countless setbacks.

#### Academic Medicine: Questioning My Voice’s Worth

***Becoming faculty*** at the heart of military medicine provided an opportunity to lead and support trainees with similar origin stories. I was excited to learn more about academic medicine and advance as faculty, but I soon realized my portfolio had a huge gap: research and publication. I actively sought collaboration opportunities, routinely discussing ideas for publication with potential mentors, but this experience was unsuccessful and demoralizing/disheartening, nothing materialized, leaving me to again question if I belong. Eventually seeing similar ideas brought to fruition in journals, I felt unsupported and invalidated. These experiences and others left me unsure of the worth of my voice in academia.

#### Mentoring and Being Mentored: I Am More than Enough

Mentoring others eventually helped me realize I had something to give. Mentoring and empowering medical students led me to addressing racism in medicine, re-defining myself as an author. Through personal and professional growth, I have realized that I am more than enough and belong in all the spaces I care to be in. “Black woman” is a broad label that masks countless ethnicities and identities. My pride and strength in being a Haitian American is masked by this label and all the racism attached to it. I still struggle to share my voice and be my full self.

## DISCUSSION

Our IM analysis using QuantCrit tools sought to create a statistical account of Black* women in medical education publishing to supplement experiential accounts. To begin, Black* women are eclipsed by white women and men in the published medical education literature. This supports Johnson’s and Karvonen et al.’s accounts about their publishing experiences.^5,6^ Further, Author 1’s testimonio demonstrates the experiential effects of this statistical account, emphasizing her struggle for belonging in academic medicine. The exclusion of Black* women’s voices and the experiential effects of that exclusion denies them equal access to the resources to create knowledge. This is something any leader in medical education publishing must take action to address. Authors 2, 3, and 5 are editors contributing to this exclusion. Following Haynes’ focus on institutional change, we commit to partnering with Black* women within our own editorial work through actions such as tracking Black* women’s publication trends, actively soliciting manuscripts from Black women authors, or intentionally amplifying and sponsoring Black* women in our field to publish. We call for others to do the same.

Furthermore, this study found that none of the top 10 MeSH topics common to Black* women authors appeared within the top 10 overall ranking of topics in medical education. In fact, the two topics Black* women authors most addressed were career mobility (defined as “the upward or downward mobility in an occupation or the change from one occupation to another”^44^) and cultural diversity (described as the “coexistence of numerous distinct ethnic, racial, religious, or cultural groups within one social unit, organization, or population”—notably, a parent term for diversity, equity, inclusion^45^). Author 1’s testimonio further contextualizes this: her entry into medical education publishing was on the topic of diversity. The focus on these topics raises questions about potential gendered/racialized topic expectations, resulting in an additional minority tax levied upon Black* women authors as “Superwomen.”^1,2^ Researchers’ topic selection behaviors are influenced by multiple intersecting factors, including gender, race/racism, research interests and background, and funding opportunities.^46^ However, research topic selection comes at a cost, with topics covered by Black* women authors garnering fewer citations compared to other groups.^17^ These findings have critical implications for Black* women researchers’ career success and trajectory and harms the field without the full and unfettered participation of Black* women.

### Limitations

First, our methodology essentialized and simplified gender and race/racism, so we could not fully instantiate IM. Racial and gender identification is manifold and dynamic (e.g., two or more races/racisms; nonbinary or shifting gender identity); applying an aggregate-level algorithm likely resulted in misattribution of racial and gender categories. Second, data were collected only for researchers identifying as based at US institutions, posing interpretation issues for global authors in the sample. Additionally, some authors may not have been born in the US, impacting SSA data, which resulted in over 1000 missing name values. Third, we used the MEJ-24, which does not include medical education articles published in journals not specific to medical education (e.g., clinical journals). Moreover, we were reliant on what data publishers—largely not Black* women—chose to collect and how they collected it.

This study suggests that the harm to Black* women in the broader field of medical education4 holds in publishing as well. Authorship—the “coin of the realm” in academia^47^—is a central piece of this puzzle. Quantitative accounts like this one help us to understand the scope of the harm. Yet we must also draw other Black* women—those who already are and desire to be authors—into dialogue through experiential accounts. How do they experience authorship in medical education and how can we ensure that they do not feel, as Johnson did amidst peer review, that “‘*you* are not enough,’ ‘your *story* is not enough,’ ‘your *voice* is not *enough*.’”?^4^
